# A Comparative Study of the Adsorption of Methylene Blue onto Synthesized Nanoscale Zero-Valent Iron-Bamboo and Manganese-Bamboo Composites

**DOI:** 10.3390/ma7064493

**Published:** 2014-06-12

**Authors:** Solomon E. Shaibu, Folahan A. Adekola, Halimat I. Adegoke, Olushola S. Ayanda

**Affiliations:** 1 Department of Chemistry, University of Ilorin, P.M.B 1515, Ilorin 240222, Nigeria; E-Mails: shaibusolomon@gmail.com (S.E.S.); faadekola@yahoo.fr (F.A.A.); ihalimat@yahoo.com (H.I.A.); 2 Environmental and Nano Science Research Group, Department of Chemistry, University of the Western Cape, Private Bag X17, Bellville 7535, South Africa

**Keywords:** bamboo, nanoscale zero-valent iron (nZVI), nanoscale manganese (nMn), methylene blue dye, adsorption

## Abstract

In this study, bamboo impregnated with nanoscale zero-valent iron (nZVI) and nanoscale manganese (nMn) were prepared by the aqueous phase borohydride reduction method and characterized using scanning electron microscopy (SEM), Fourier transform infrared spectroscopy (FTIR) and PIXE analysis. The synthesized nMn-bamboo and nZVI-bamboo composites were subsequently applied to the sorption of methylene blue (MB) dye from aqueous solution. The adsorption of MB dye was investigated under various experimental conditions such as pH, contact time, initial concentration of MB dye and adsorbent dosage. The results showed that the synthesized nZVI-bamboo composite was more effective than nMn-bamboo composite in terms of higher MB dye adsorption capacity of 322.5 mg/g compared to 263.5 mg/g of nMn-bamboo composite. At a concentration of 140 mg/L MB dye, 0.02 g of nZVI-bamboo and nMn-bamboo composites resulted in 79.6% and 78.3% removal, respectively, at 165 rpm, contact time of 120 min and at a solution pH of 7.6. The equilibrium data was best represented by Freundlich isotherm model and the pseudo-second order kinetic model better explained the kinetic data for both nZVI-bamboo and nMn-bamboo composites.

## 1. Introduction

Rapid industrialization and urbanization have resulted in the generation of large quantities of aqueous effluents, many of which contain high levels of toxic pollutants such as dyes, persistent organic pollutants (POPs), heavy metals *etc.* [1,2]. Dyes are highly colored polymers with low biodegradability and are highly recalcitrant, persist for long distances in flowing water, retard photosynthetic activity, inhibit the growth of aquatic biota by blocking out sunlight and utilizing dissolved oxygen, and also decrease the recreation value of stream. Almost every industry uses coloring matter to color their products. The total dye consumption of the textile industries alone is in excess of 10^7^ kg/year and an estimated 90% of this total ends up on fabric [3]. Consequently, approximately 106 kg/year of dyes are discharged into the waste streams by textile industries. Dye industry effluents constitute one of the most problematic wastewaters to be treated not only for their high chemical and biological oxygen demands (BOD) and suspended solid and content in the toxic compounds but also for their aesthetic impact [4]. Over the years, various methods of wastewater treatment have been reported and adsorption is one of the most widely accepted techniques in terms of its cost effectiveness, versatility, simplicity and ease of operation [5,6,7]. The use of nanoscale composite materials has been reported over the years by quite a number of researchers for the treatment of wastewater. Some include the remediation of tributyltin using nFe_3_O_4_, activated carbon and nFe_3_O_4_/activated carbon composite material reported by Ayanda *et al.* [8], the synthesis of supported nanoscale zero-valent iron (nZVI) was reported by Zhang *et al.* [9] where nZVI supported on exfoliated graphite was used for the removal of nitrate. Sheela *et al.* [10] and Rahmani *et al.* [11] investigated the adsorption of heavy metals onto nanoscale zinc oxide (nZnO) and alumina, respectively. In addition, Frost *et al.* [12] used palygorskite supported zero-valent iron for the removal of methylene blue (MB) and Shahryari *et al.* [13] reported the adsorption of MB onto carbon nanotubes. However, no work has been reported on the use of nZVI- and nanoscale manganese (nMn)-bamboo composites for the removal of MB from aqueous solution. Bamboo is a low cost agricultural waste material, readily available, and its composition with nZVI and nMn will improve some of its physicochemical properties and also serve as a means for conversion of the agricultural waste (bamboo) to economical use, through its application in the removal of MB. Moreover, bamboo supported nZVI because of the need to overcome some of the disadvantages inherent in the use of nZVI which reduces its efficiency and usability, such as rapid oxidation, a strong tendency to agglomerate into larger particles and also the separation and recovery of the fine particles after usage.

The objective of this research is to evaluate the effectiveness of synthesized nZVI-bamboo and nMn-bamboo composites for the removal of MB as a model compound for basic dyes. The effects of pH, contact time, initial dye concentration and adsorbent dosage on adsorption capacity will be investigated. In the same vein, equilibrium and kinetic models will be used to fit the experimental data and the equilibrium and kinetic constants determined. Results from this study can be used to assess the utility of nZVI-bamboo and nMn-bamboo composites for dyes removal, in particular MB adsorption, at the field scale.

## 2. Results and Discussion

### 2.1. Scanning Electron Microscopy (SEM)

**Figure 1:**
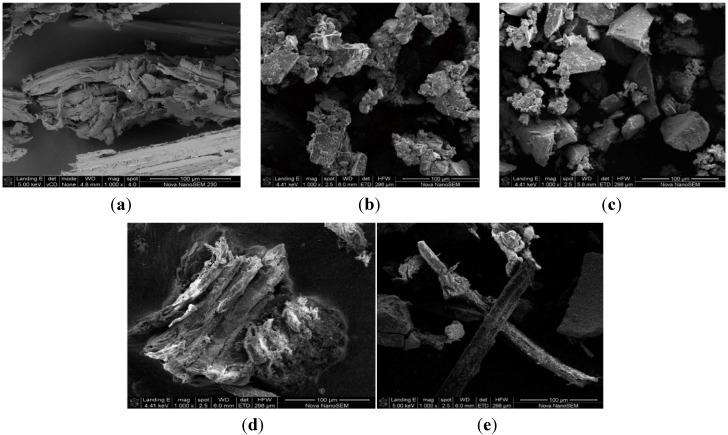
SEM images of (**a**) Bamboo powder (mag 5000×); (**b**) nZVI (mag 1000×); (**c**) nMn (mag 1000×); (**d**) nZVI-bamboo composite (mag 1000×); and (**e**) nMn-bamboo composite (mag 1000×).

The SEM images of the synthesized nZVI, nMn, bamboo, nZVI-bamboo and nMn-bamboo composites are presented in Figure 1. The SEM image of nZVI in Figure 1b showed agglomeration of the iron nanoparticles, the size of which might be in micron rather than existing as separate nanoparticles. This is similar to what was earlier reported by Çelebi *et al.* [14], and as can be observed from the SEM images, the synthesized nZVI and nMn particles in Figure 1b,c respectively, were aggregated which is caused by the large surface area and magnetic dipole–dipole interactions of the individual particles as reported by Li *et al.* [15]. 

The micrographs of the bamboo and nZVI-bamboo are also shown in Figure 1a,d, respectively, and aggregation was also observed in the nZVI-bamboo composite due to synthesis at ambient conditions which is also similar to that observed in nMn-bamboo composite in Figure 1e. In addition, the SEM image of nZVI-bamboo composite similar to that of nMn-bamboo composite (Figure 1d,e) showed that the bamboo fibre was completely surrounded by the nanoparticles. The nMn particles in Figure 1c were evenly distributed on the surface of the bamboo fibres, thereby increasing the surface area of the nMn-bamboo composite. Also, the pores and crevices on the surface of the bamboo fibre have been filled with the nanoscale iron and manganese particles due to their comparatively smaller sizes.

### 2.2. Fourier Transform Infrared Spectroscopy (FTIR)

The FTIR spectra of bamboo, nZVI, nMn, nZVI-bamboo and nMn-bamboo are presented in the supplementary document, Figures S1–S5. The FTIR spectra of bamboo show a strong broad O–H stretching absorption at 3417.98 cm^−1^ and a prominent C–H stretching absorption around 2939.61 cm^−1^. The non-conjugated C–O stretch (in hemicellulose) was observed at 1728.28 cm^−1^, and the aromatic skeletal vibration in lignin appeared at 1604.83 cm^−1^. There was also a stronger carbonyl band at 1728.28 cm^−1^, indicating relatively high-xylan content present in bamboo. Due to the composition of nMn and nZVI with the bamboo, some prominent peaks could be seen among the absorption bands of nMn-bamboo and nZVI-bamboo composites, such as those at 3441.12 cm^−1^, 1728.28 cm^−1^, 1639.55 cm^−1^, 1604.83 cm^−1^, due to O–H stretching, non-conjugated C–O, conjugated C=O in lignin, and aromatic skeletal vibration in lignin, respectively. The FTIR spectrum of nZVI-bamboo composite is similar to that of nMn-bamboo composite except for some little band differences as shown in Table 1.

**Table 1:** Diagnostic bands of bamboo, nMn, nMn-bamboo, nZVI, and nZVI-bamboo composite.

Bamboo (cm^−1^)	nMn (cm^−1^)	nMn-Bamboo (cm^−1^)	nZVI (cm^−1^)	nZVI-Bamboo (cm^−1^)
3417.98	3404.47	3417.98	3421.83	3441.12
2939.61	–	2895.25	–	2899.11
1728.28	–	1726.35	–	1728.28
1604.83	1629.9	1604.83	1637.62	1639.55

### 2.3. Particle Induced X-ray Emission (PIXE)

The elements present ranging from Si to Zn are shown in Table 2. The concentration of some metals in nMn-bamboo composite, such as Al, Si, Ti, Zn, Ni, and Mn decreased while those of K, Ca and Fe were higher compared to that of nMn while in the case of nZVI-bamboo composite, an increase was observed in the concentration of Al, K, Ca, Cr and Zn which might be due to the concentrations of FeCl_3,_ MnCl_4_·H_2_O and NaBH_4_ used in the preparation of the composites. Chromium (Cr) and nickel (Ni) were absent in nMn-bamboo and nZVI-bamboo composites, respectively.

**Table 2:** Concentration of elements in bamboo, nMn, nMn-bamboo, nZVI, and nZVI-bamboo composite by PIXE analysis.

Symbol	Concentration (mg/L)				
	Bamboo	nMn	nMn-Bamboo	nZVI	nZVI-Bamboo
Al	4696.8	23,563.1	7369.7	10,785.7	12,088.1
Si	3047.1	8755.9	3485.9	12,516.6	2161.0
Cl	289.1	64,805.2	46,723.5	20,680.3	16,791.6
K	72.3	nd	62.6	76.0	79.8
Ca	110.6	261.1	345.4	335.0	598.8
Ti	9.5	60.8	35.4	123.9	89.8
Cr	13.0	nd	nd	68.8	83.5
Mn	37.5	336,339.4	109,879.9	1584.7	747.7
Fe	363.8	3889.4	4232.2	331,691.0	179,229.8
Ni	nd	320.1	48.0	nd	nd
Zn	7.3	155.4	93.4	nd	2.4

nd—not detected (represents concentration that are below the detection level of the machine).

### 2.4. Effect of Initial Concentration

**Figure 2:**
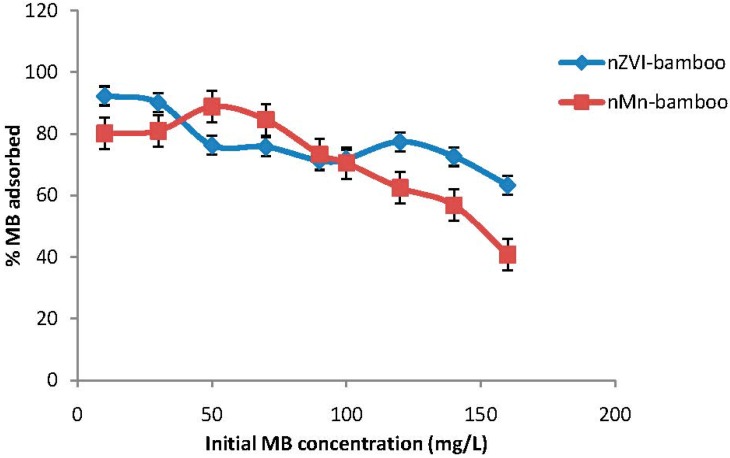
Effect of initial methylene blue (MB) concentration (*wt* = 0.02 g, pH = 5, *T* = 32 ± 2 °C for 2 h at 165 rpm).

The initial concentration provides an important driving force in order to overcome all mass transfer resistance of the dye between the aqueous and the solid phase. The percent removal of MB for both composite materials decreases with an increase in initial concentration as shown in Figure 2, suggesting that at lower MB concentration, there were many vacant adsorption sites available for the MB molecules to attach to until the surface of the adsorbents was saturated at 140 mg/L where the amount of MB adsorbed did not increase significantly. In addition, the percentage removal of MB dye decreased from 92.3% to 63.3% using nZVI-bamboo composite while that of nMn-bamboo composite decreased from 80.1% to 40.8% as the initial concentration of MB dye increased from 10 to 160 mg/L. Similar findings were reported by Shirmadi *et al.* [16].

### 2.5. Effect of Adsorbent Dosage

**Figure 3:**
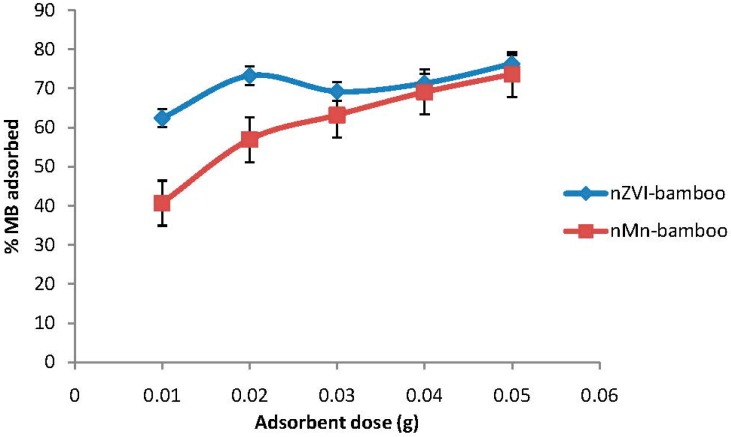
Effect of adsorbent dosage (*C*_0_ = 140 mg/L, pH = 5, *T* = 32 ± 2 °C for 2 h at 165 rpm).

The amount of available surface area under an effectively constant metal surface is one of the most significant experimental variables affecting contaminant reduction rate. The effect of adsorbent dosage on MB sorption is shown in Figure 3 and it followed the predicted pattern of an increasing percentage adsorbed as the dosage of the different adsorbents increases. The removal of MB increased from 62.4% to 76.3% and 40.7% to 73.7% for nZVI-bamboo and nMn-bamboo composites, respectively, which could be as a result of an increase in the surface area and availability of more adsorption sites as the dosage increased, which is similar to the report of Cengiz and Cavas [17].

### 2.6. Effect of Initial pH

**Figure 4:**
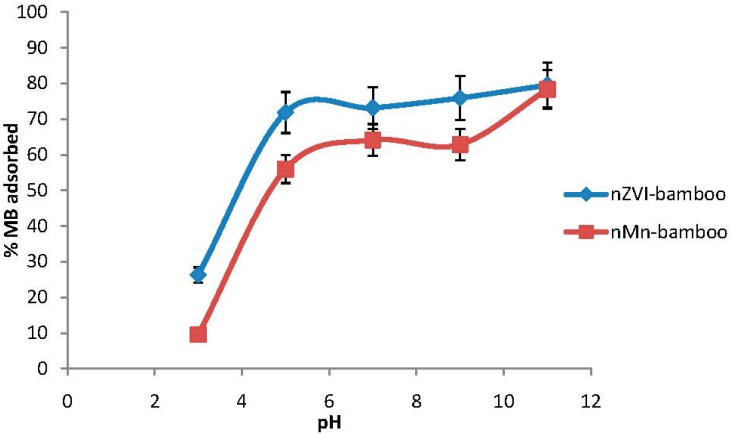
Effect of pH (*wt* = 0.02 g, *C*_0_ = 140 mg/L, *T* = 32 ± 2 °C for 2 h at 165 rpm).

The solution pH would affect both aqueous chemistry and surface binding sites of the adsorbents. So, the pH is an important parameter in the dye adsorption process [18]. The hydrogen ion concentration (pH) primarily affects the degree of ionization of the dyes and the surface properties of the adsorbent. As can be seen from Figure 4, the adsorption of MB dye onto nZVI-bamboo and nMn-bamboo composites is highly dependent on the initial pH of solution. Higher removal percentages of MB dye from solution was observed for nZVI-bamboo and nMn-bamboo composites at pH 11, being 79.6% and 78.3%, respectively. The adsorption capacity of MB increased with increasing solution pH from 3 to 5. No significant increase was further noted until pH 9 where a rapid adsorption was observed. This result can be attributed to the effect of the solution pH on the charge of reactive group within the adsorbents which in turn makes them more effective at adsorbing MB dye in alkaline pH. A similar observation was reported by Hu *et al.* [19] and Salman *et al.* [20]. 

### 2.7. Effect of Time

**Figure 5:**
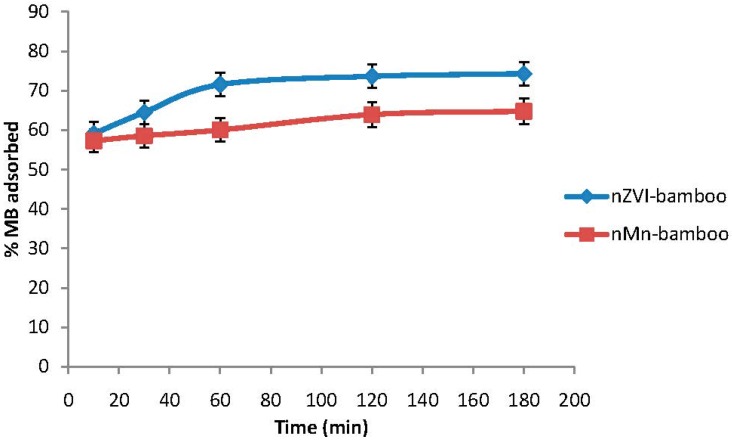
Effect of time (*wt* = 0.02 g, pH = 5; *C*_0_ = 140 mg/L, *T* = 32 ± 2 °C at 165 rpm).

The graph of the amount of MB adsorbed *versus* contact time is represented in Figure 5. It revealed that the MB adsorption was fast at the initial stage of the contact period and then became slower near the equilibrium time (120 min) which is similar to the result reported by Karima *et al.* [21]. This phenomenon was due to the fact that a large number of vacant surface sites were available for adsorption during the initial stage of the adsorption process. Near the equilibrium, the remaining vacant surface sites were difficult to occupy due to, probably, the slow pore diffusion of the MB molecules on the adsorbents (nZVI-bamboo and nMn-bamboo) and the repulsive forces between the solid molecules and the bulk phases. 

### 2.8. Adsorption Isotherm

The results of the MB concentration dependence study were subjected to analyses by means of Langmuir, Freundlich and Temkin adsorption isotherm models. The Langmuir equation (Equation (1)), which is valid for monolayer sorption on a surface containing a limited number of sites, predicting a homogeneous distribution of sorption energies and the heterogeneity of adsorption deduced from Freundlich equation (Equation (2)). Temkin model (Equation (3)) assumes that heat of adsorption (function of temperature) of all molecules in the layer would decrease linearly rather than logarithmically with coverage.

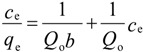
(1)

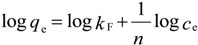
(2)

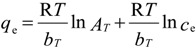
(3)
where *C*_e_ is the equilibrium concentration of the adsorbate (mg/L), *q*_e_ the amount of adsorbate adsorbed per unit mass of adsorbate (mg/g), *Q*_o_ and *b* are Langmuir constants related to adsorption capacity and rate of adsorption, respectively. *k*_F_ and n are Freundlich constants, an indication of how favorable the adsorption process is and *k*_F_ is the adsorption capacity of the adsorbent, *A_T_* is Temkin isotherm equilibrium binding constant (L/g), *b_T_* is Temkin isotherm constant (J/mol), R is universal gas constant (8.314 J/mol/K) and *T* is the absolute temperature.

**Figure 6:**
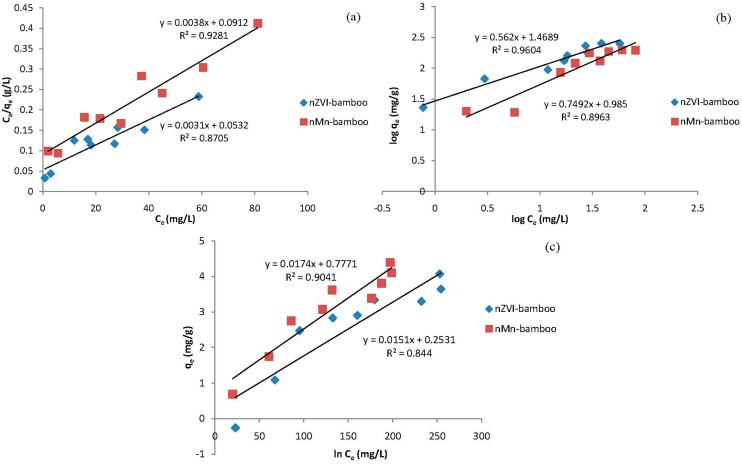
(**a**) Langmiur; (**b**) Freundlich; and (**c**) Temkin Isotherms for the adsorption of MB onto nZVI-bamboo and nMn-bamboo composites.

The adsorption data of nZVI-bamboo and nMn-bamboo composites were adequately interpreted by Freundlich and Langmuir isotherms, respectively based on their regression coefficient (Figure 6 and Table 3). 

**Table 3:** Isotherm parameters for the adsorption of MB onto nMn-bamboo and nZVI-bamboo composites.

Isotherm	Parameters	Values	
		(nMn-bamboo)	(nZVI-bamboo)
Freundlich	*K*_F_ (mg/g(L/mg)^1/*n*^)	9.66	29.4
	*n*	1.33	1.78
	*R*^2^	0.8963	0.9604
Langmuir	*Q*_o_ (mg/g)	263.2	322.6
	*b* (L/mg)	0.0417	0.0583
	*R*_L_	0.13	0.097
	*R*^2^	0.9281	0.8705
Temkin	*A_T_* (L/g)	1.69	1.20
	*b_T_* (J/mol)	43.9	40.7
	*R*^2^	0.9041	0.844

The adsorption is evidently favorable from the value of the Freundlich constant *n* which is 1.901 and 1.335 while the constant *k*_F_ has a value of 29.437 and 9.66 for nZVI-bamboo composite and nMn-bamboo, respectively, indicative of the affinity of the MB dye and the composite materials. The good agreement of Langmuir’s isotherm with the adsorption data may be due to homogeneous distribution of active sites on the nMn-bamboo composite, since the Langmuir equation (Equation (1)) assumes that the surface is homogeneous. The values of the adsorption coefficient b and the monolayer capacity *Q*_o_ calculated from Langmuir equation (Equation (1)) is given in Table 3. 

To know the feasibility of this isotherm, the essential features of Langmuir model can be expressed in terms of R_L_. The values of R_L_ indicate the shapes of isotherms to be either unfavorable (*R*_L_ > 1), linear (*R*_L_ = 1), favorable (0 < *R*_L_ < 1) or irreversible (*R*_L_ = 0) [22]. The calculated *R*_L_ values (0.13 and 0.096) show that the adsorption of MB onto nZVI-bamboo and nMn-bamboo composite is favorable.

Table 4 shows the saturated adsorption capacities of MB for ZVI-bamboo and nMn-bamboo composites along with some other adsorbents in literature. It could be clearly seen that nZVI-bamboo and nMn-bamboo composites exhibited higher saturated adsorption capacity, which could be ascribed to their larger surface area and numerous pores compared to other adsorbents listed in Table 4.

**Table 4:** Saturated adsorption capacities of MB for some adsorbents.

Adsorbent	Saturated Adsorption Capacity (mg/g)	References
Zeolite	16.37	[23]
CTNTs	133.33	[24]
Activated sewage char	120.00	[25]
Raw date pits	80.29	[26]
nMn-bamboo	263.2	Present work
nZVI-bamboo	322.6	Present work

### 2.9. Kinetic Models

To study the kinetics of the adsorption of MB onto nZVI-bamboo and nMn-bamboo composites, three different kinetic models were used for the analysis of the adsorption data of each of the adsorbents, viz: pseudo-first order (Equation (4)), pseudo-second order (Equation (5)) and Elovich (Equation (6)) models.

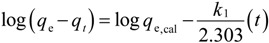
(4)

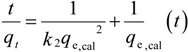
(5)

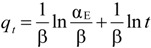
(6)
where *k*_1_, *k*_2_, α_E_, and β, are the pseudo first-order, pseudo second-order and Elovich constants, respectively. 

**Figure 7:**
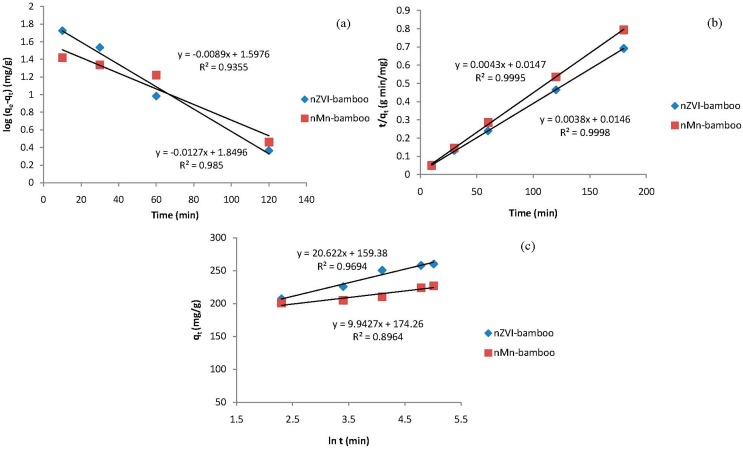
(**a**) Pseudo-first order; (**b**) pseudo-second order; and (**c**) Elovich models for the adsorption of MB onto nZVI-bamboo and nMn-bamboo composites.

Evidently, the adsorption data fitted the pseudo-second order kinetic model with regression coefficients of 0.9995 and 0.9998 for nZVI-bamboo and nMn-bamboo composites, respectively than pseudo-first order and Elovich models which also show a fairly good interpretation of the adsorption data as shown in Figure 7 and Table 5.

Experimental results thus revealed that the support (bamboo) stabilizes the nZVI and nMn particles and the mass transfer of MB to nZVI surfaces was promoted because of the adsorption by the support. These prevent the agglomeration of nZVI during adsorption and eventually enhance the efficiency of these materials to remove MB dyes [27,28]. Moreover, bamboo is readily available in our environment as waste while nanoparticles are expensive due to various instrumental techniques that are required to inform us about the properties of the synthesized nanoparticles. A combination of bamboo with nanoparticles will reduce the cost of using only nanoparticles for the remediation of organic pollutants and will lead to improvements in the physicochemical properties of the formed composite.

**Table 5:** Parameters and correlation coefficients of the kinetic models for nMn-bamboo and nZVI-bamboo composites.

Kinetic Models	Parameters	Values	
		(nMn-bamboo)	(nZVI-bamboo)
Pseudo-first order	*k*_1_ (min^−1^)	0.0205	0.0290
	*q*_e,cal_ (mg/g)	39.6	70.7
	*R*^2^	0.9350	0.9850
Pseudo-second order	*k*_2_ (g/mg/min)	0.0013	0.0009
	*q*_e,cal_ (mg/g)	232.56	263.16
	*R*^2^	0.9995	0.9998
Elovich	β (g·min/mg)	0.0485	0.1006
	α_E_ (g·min^2^/mg)	4.69 × 10^3^	4.09 × 10^8^
	*R*^2^	0.8964	0.9694

## 3. Experimental Section

### 3.1. Reagents

Analytical grade iron (III) chloride (Sigma Aldrich, Diegem, Belgium), manganese (IV) chloride (Minimum assay 99.9%, Tianjin Kermel, Hebei, China), sodium borohydride (BDH 95%, prd No. 30114, Sigma Aldrich, Diegem, Belgium), MB dye (BDH prd No. 340484B, Sigma Aldrich, Diegem, Belgium), absolute ethanol (BDH Analar, 95% UN No. 1097, Sigma Aldrich, Diegem, Belgium), hydrochloric acid (Purity 37%, density 1.1 kg/cm^3^, Riedel-deHaen, Buffalo, NY, USA) and sodium hydroxide (BDH prod. No. 30167, Sigma Aldrich, Diegem, Belgium) were used without further purification. Other basic laboratory glass-wares and apparatus were also used in the course of this research.

### 3.2. nZVI-Bamboo Composite Preparation

The nZVI-bamboo composite was prepared by borohydride reduction method with FeCl_3_ and bamboo in the ratio 1:1. The reduction method was related to the method reported by Yuvakkumar *et al.* [29]. A 0.8111 g of treated bamboo (*i.e.*, the bamboo was washed with 0.1 M HCl solution, dried and ground into fine powder) was weighed and added into a solution of 0.05 M FeCl_3_ in a beaker and stirred on a magnetic stirrer for 2 h to ensure thorough mixture of both materials and then poured into a three neck flask before addition of the borohydride solution. A 0.53 M NaBH_4_ solution was added into the beaker containing the mixture of the bamboo and FeCl_3_ solution. After the complete addition of the borohydride solution, the resulting mixture was further stirred for another 30 min. The resulting precipitate was washed several times with ethanol and filtered through 0.45 µm millipore filter paper by vacuum filtration technique and was oven dried at 50 °C overnight [12].

### 3.3. nMn-Bamboo Composite Preparation

**Figure 8:**
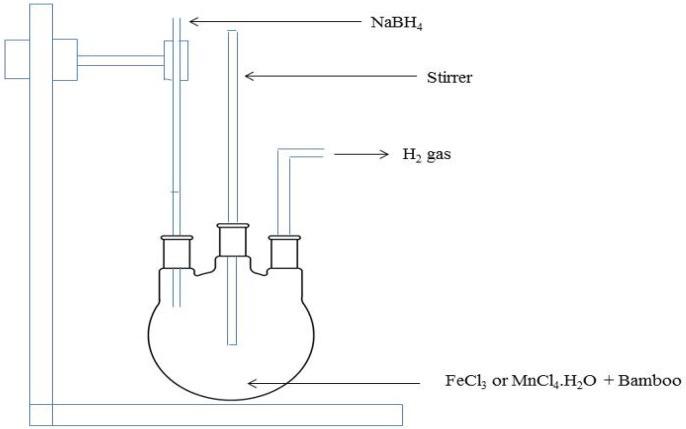
Schematic diagram for the synthesis of nZVI-bamboo and nMn-bamboo composites.

The nMn-bamboo composite was also prepared by borohydride reduction method similar to that of nZVI-bamboo composite with MnCl_4_·H_2_O and bamboo in the ratio 1:1. A 1.979 g of the treated bamboo was weighed and added into a solution of 0.05 M MnCl_4_·H_2_O in a beaker and stirred on a magnetic stirrer for 2 h to ensure thorough mixture of both materials and then poured into a three neck flask before addition of the borohydride solution. A 0.53 M NaBH_4_ solution was added into the beaker containing the mixture of the bamboo and MnCl_4_·H_2_O solution. After the complete addition of the borohydride solution, the resulting mixture was further stirred for another 30 min. The resulting precipitate was subjected to similar filtration and drying process as that of nZVI-bamboo composite. Figure 8 shows the schematic diagram for the synthesis of nZVI-bamboo and nMn-bamboo composites.

### 3.4. Characterization of nZVI-Bamboo and nMn-Bamboo Composites

The scanning electron micrograph (SEM) of nZVI, nMn, bamboo, nZVI-bamboo and nMn-bamboo composites was viewed under a FEI^TM^ scanning electron microscope (Nova Nano SEM 230, FEI, Hillsboro, OR, USA). A 1.7 MV 5SDH tandem pelletron accelerator was used to analyze for the concentration of the elements present. Fourier transform infrared spectroscopy (FTIR, Shimadzu, Columbia, MD, USA) absorption spectra were obtained using the potassium bromide (KBr) pellet method and the spectra of the samples were recorded over the range 4000–400 cm^−1^ using Shimadzu FTIR-8400S.

### 3.5. Adsorption Studies

Dye removal experiments with the synthesized nZVI-bamboo and nMn-bamboo composites were carried out by batch tests in 100 mL flasks under vigorous agitation at 165 rpm. The experiment involved preparing 50 mL of MB solution with a desired initial concentration (140 mg/L) and pH, by diluting the stock dye solutions with deionized water and transferring it into the conical flask for agitation. The pH of the solution was adjusted using 0.1 M HCl or 0.1 M NaOH solutions by a pH meter model (pH 211 Microprocessor). A 0.02 g of nZVI-bamboo and nMn-bamboo composites (adsorbent dosage) was then added to different conical flask containing dye solution and the obtained suspensions were immediately agitated for a predefined time of 120 min. After the contact time elapsed, the different suspensions were filtered separately and the filtrates were analyzed using a UV/visible spectrophotometer (UV/vis DU 730, Beckman Coulter, Pasadena, CA, USA) at maximum absorption wavelength of 665 nm for MB dye. The amount of MB adsorbed, *q*_e_ (mg/g) was obtained using Equation (7):

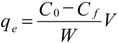
(7)
where *C_0_* and *C_f_* are the initial and final concentrations of MB dye (mg/L), respectively. *V* is the volume of the solution (L) and *W* is the mass of adsorbents (g). The % adsorbed was calculated using Equation (8).

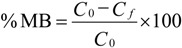
(8)


All tests were performed in triplicate to ensure the reproducibility of the results; the mean of the measurements was reported. Furthermore, all experiments were performed at room temperature (32 ± 2 °C). The investigated ranges of the experimental variables were as follows: MB dye concentration (10–160 mg/L), initial pH of solution (3–11), adsorbent dosage (0.01–0.05 g) and contact time of 10–180 min.

## 4. Conclusions

This study confirmed that the nZVI-bamboo and nMn-bamboo composites prepared by the borohydride reduction method were effective for the removal of MB dye from aqueous solution. The results showed that the adsorption is highly influenced by the initial concentration, solution’s pH, adsorbent dosage and contact time. The adsorption data were adequately interpreted by Freundlich and Langmuir adsorption isotherm for nZVI-bamboo and nMn-bamboo composites, respectively, and the kinetic data were better explained by the pseudo-second order kinetic model for both composite materials. The results of this experimental study are highly useful for the remediation of real industrial effluent or MB dye-laden wastewater.

## Supplementary Materials

Supplementary File 1
