# Supplementary material for: A Comparative Study of the Adsorption of Methylene Blue onto Synthesized Nanoscale Zero-Valent Iron-Bamboo and Manganese-Bamboo Composites
Source: Materials (Basel). 2014 Jun 12;7(6):4493–507. doi: 10.3390/ma7064493 (PMC5455915; doi:10.3390/ma7064493)
Supplement: Supplementary File 1 [file materials-07-04493-s001.zip › materials-54432-supplementary.pdf]

Supplementary Materials

Highlights

- Bamboo impregnated with nanoscale zero-valent iron (nZVI) and manganese (nMn) were prepared by the aqueous phase borohydride reduction method.
- The precursors and composites were characterized by SEM, FTIR and PIXE analysis.
- Methylene blue adsorption capacity, isotherm and kinetics of these composites were investigated and compared.
- nZVI- and nMn-bamboo composites are better materials for the remediation of organic dyes from wastewaters.

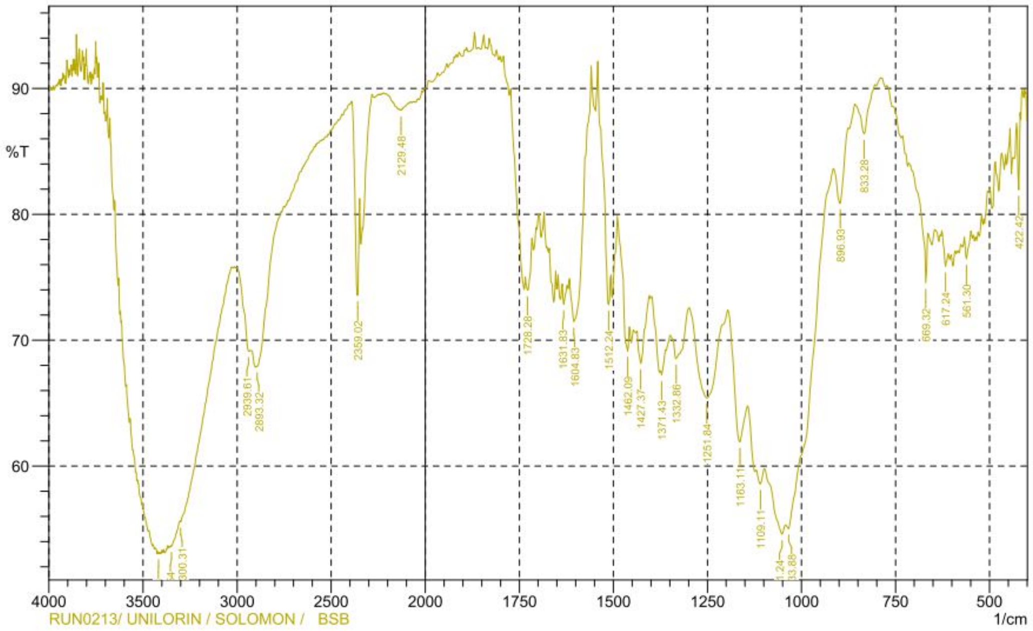

Figure S1. FTIR of Bamboo.

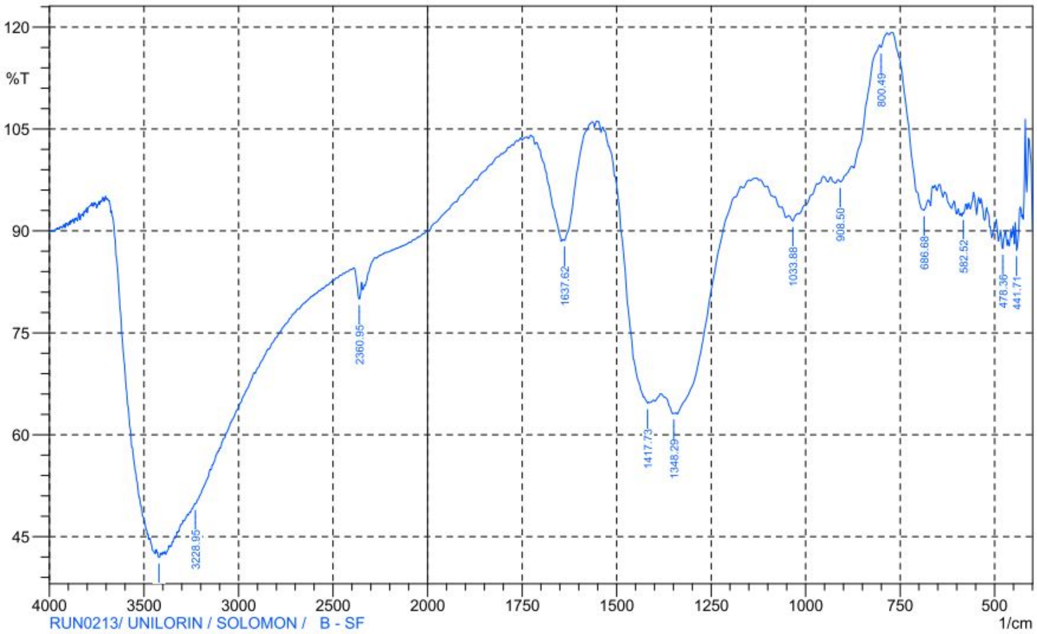

Figure S2. FTIR of nZVI.

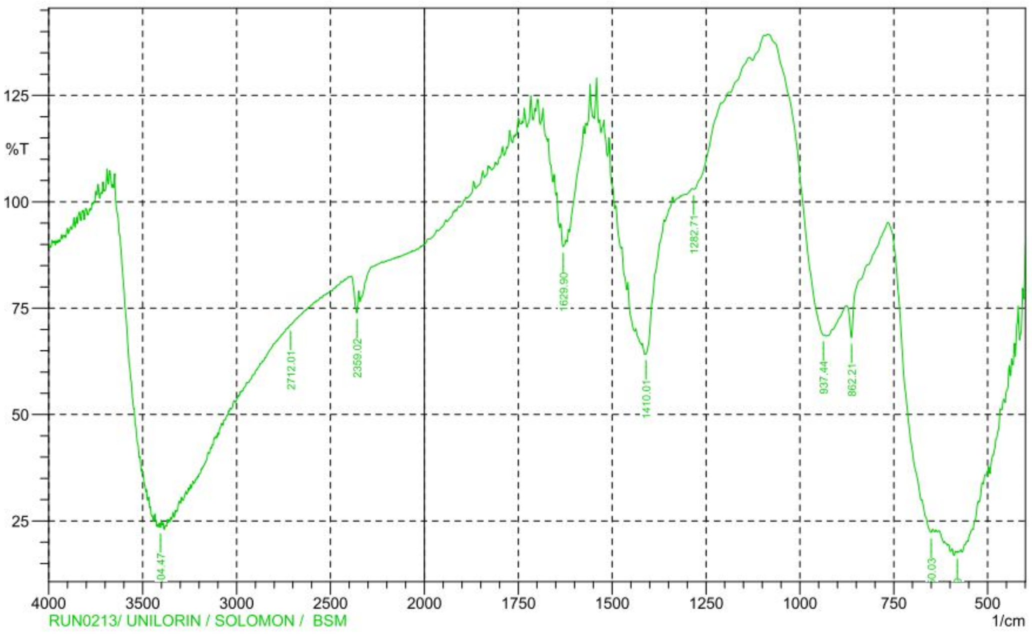

Figure S3. FTIR of nMn.

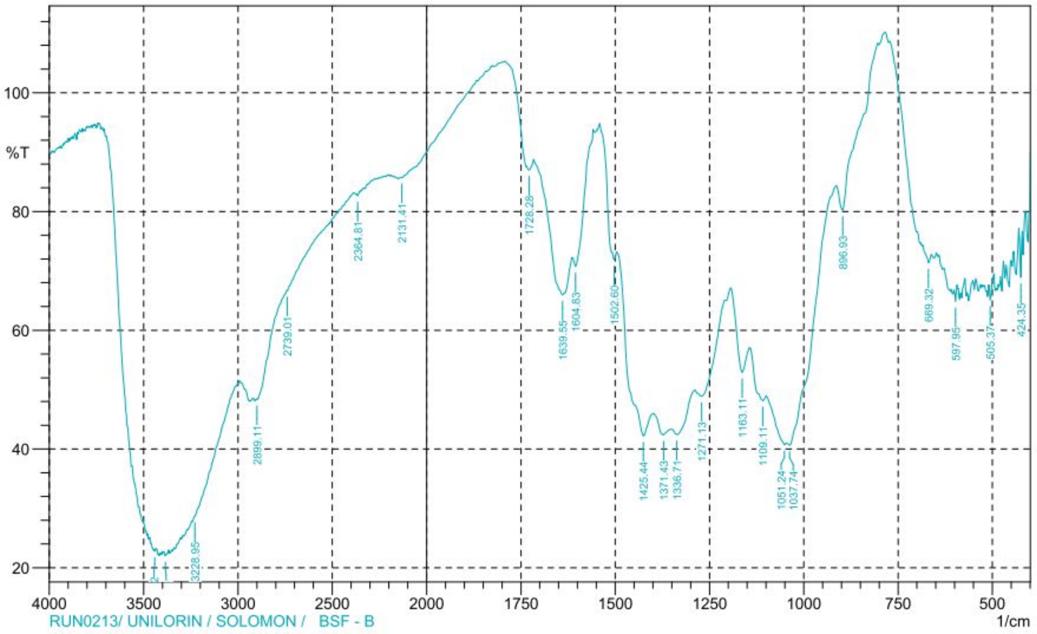

Figure S4. FTIR of nZVI-bamboo composite.

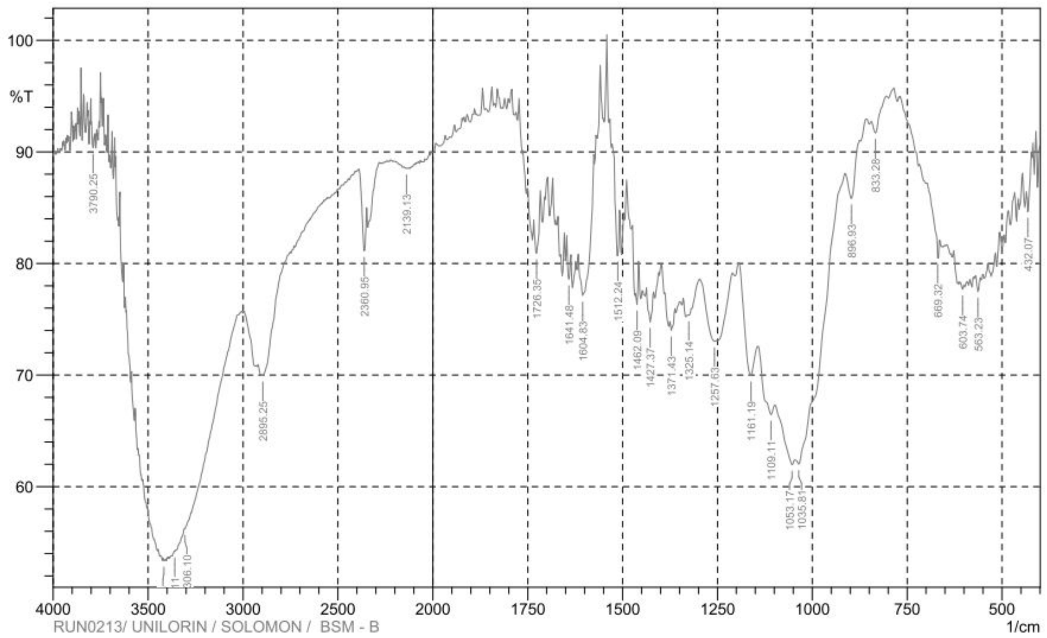

Figure S5. FTIR of nMn-bamboo composite.
